# Comparison of alternative approaches to single-trait genomic prediction using genotyped and non-genotyped Hanwoo beef cattle

**DOI:** 10.1186/s12711-016-0279-9

**Published:** 2017-01-04

**Authors:** Joonho Lee, Hao Cheng, Dorian Garrick, Bruce Golden, Jack Dekkers, Kyungdo Park, Deukhwan Lee, Rohan Fernando

**Affiliations:** 1 Department of Animal Science, Iowa State University, Ames, IA 50011 USA; 2 Department of Statistics, Iowa State University, Ames, IA 50011 USA; 3 Institute of Veterinary, Animal and Biomedical Sciences, Massey University, Palmerston North, New Zealand; 4 ThetaSolutions, LLC, Atascadero, CA USA; 5 Department of Animal Biotechnology, Chonbuk National University, Chonju, Jeollabuk-do South Korea; 6 Department of Animal Science, Hankyong National University, Anseong, Gyeonggi-do South Korea

## Abstract

**Background:**

Genomic predictions from BayesA and BayesB use training data that include animals with both phenotypes and genotypes. Single-step methodologies allow additional information from non-genotyped relatives to be included in the analysis. The single-step genomic best linear unbiased prediction (SSGBLUP) method uses a relationship matrix computed from marker and pedigree information, in which missing genotypes are imputed implicitly. Single-step Bayesian regression (SSBR) extends SSGBLUP to BayesB-like models using explicitly imputed genotypes for non-genotyped individuals.

**Methods:**

Carcass records included 988 genotyped Hanwoo steers with 35,882 SNPs and 1438 non-genotyped steers that were measured for back-fat thickness (BFT), carcass weight (CWT), eye-muscle area, and marbling score (MAR). Single-trait pedigree-based BLUP, Bayesian methods using only genotyped individuals, SSGBLUP and SSBR methods were compared using cross-validation.

**Results:**

Methods using genomic information always outperformed pedigree-based BLUP when the same phenotypic data were modeled from either genotyped individuals only or both genotyped and non-genotyped individuals. For BFT and MAR, accuracies were higher with single-step methods than with BayesB, BayesC and BayesC*π*. Gains in accuracy with the single-step methods ranged from +0.06 to +0.09 for BFT and from +0.05 to +0.07 for MAR. For CWT, SSBR always outperformed the corresponding Bayesian methods that used only genotyped individuals. However, although SSGBLUP incorporated information from non-genotyped individuals, prediction accuracies were lower with SSGBLUP than with BayesC (*π* = 0.9999) and BayesB (*π* = 0.98) for CWT because, for this particular trait, there was a benefit from the mixture priors of the effects of the single nucleotide polymorphisms.

**Conclusions:**

Single-step methods are the preferred approaches for prediction combining genotyped and non-genotyped animals. Alternative priors allow SSBR to outperform SSGBLUP in some cases.

## Background

Since breeding technologies using genome-wide single nucleotide polymorphism (SNP) panels became available, genomic selection was rapidly adopted for improvement of livestock and has replaced the traditionally used pedigree-based best linear unbiased prediction (PBLUP). The BayesA and BayesB hierarchical Bayesian models with locus-specific variances were proposed by Meuwissen et al. [1]. BayesB can accommodate mixture models in which SNPs have zero effects with probability *π* [2, 3]. When *π* = 0, BayesB is known as BayesA. BayesC is another widely-used Bayesian mixture model, in which a common variance is used for all SNPs instead of locus-specific variances [4], and a modification of that method known as BayesC*π* treats *π* as an unknown parameter with a uniform prior distribution [5].

In general, the number of individuals with genomic information is a small subset of the individuals represented in the population with pedigree and phenotypic information. “Single-step” methodologies were developed to take advantage of all pedigree, phenotypic and genomic information simultaneously [6, 7]. The single-step genomic BLUP (SSGBLUP) method uses a relationship matrix that is computed from marker and pedigree information. SSGBLUP was shown to yield a similar or higher accuracy compared to methods using only genotyped individuals [8–10]. Fernando et al. [7] proposed a class of single-step Bayesian regression methods (SSBR) to extend SSGBLUP to incorporate BayesB-like models for SNP effects (SSBR-B). Similar extensions of SSGBLUP with BayesC-like models result in SSBR-C and SSBR-C*π*. SSBR methods may promise higher prediction accuracies and provide computational benefits when many animals are genotyped. In SSGBLUP, the distribution of marker effects conditional on the variance of marker effects is assumed univariate normal, whereas in SSBR, the prior for marker effects can follow a t-distribution, a double exponential distribution or mixture distributions, which may be advantageous in some situations.

In this paper, prediction accuracies from PBLUP, BayesB, BayesC, BayesCπ, SSGBLUP and SSBR-B, SSBR-C, SSBR-C*π* were compared in terms of cross-validation accuracies.

## Methods

### Data

Young Hanwoo bulls are routinely progeny-tested in batches at the Hanwoo Improvement Center (Seo-San, Chungnam, South Korea). DNA samples were collected from steers that included the progeny-tested offspring from the 46th to 51st selection batches. SNP genotypes were determined using Illumina Bovine SNP50 v1 (50 k) or Bovine HD (778 k) beadchips (Illumina, CA).

Carcass records were recorded at harvest at about 24 months of age. The carcass traits used in the analyses were back-fat thickness (BFT), carcass weight (CWT), eye-muscle area (EMA), and marbling score (MAR). Park et al. [11] reported heritabilities of 0.50, 0.30, 0.42 and 0.63 for BFT, CWT, EMA and MAR, respectively. Approval from the ethics committee was not required for these data since they were obtained from an existing industry database.

Of the 44 k SNPs that are included on both the 50 and 778 k beadchips, only autosomal SNPs with known map location were used. For quality control, SNPs that departed from the Hardy–Weinberg equilibrium (p < 10^−6^) based on a Chi square test, or had a minor allele frequency (MAF) lower than 0.01, or a missing rate higher than 0.1 were excluded from further analysis. For the genotyped animals, SNPs with missing genotypes were imputed using Beagle 3.3 [12]. After these quality controls, 35,882 SNPs remained for analyses.

The numerator relationship matrix (NRM) based on pedigree information and the genomic relationship matrix (GRM) based on SNP genotypes were compared. Nineteen individuals, which showed unreasonable deviations between the NRM and GRM coefficients that were probably due to errors in the DNA sampling, were eliminated. Among these 19 individuals, five appeared to have been genotyped twice with different ID since their GRM relationship coefficients were near 1.0 while their NRM relationship coefficients were close to 0. For the other 14 individuals, either the GRM relationship coefficients were near 0 while those of the NRM were near 0.25 as would be the case for mistakenly recorded half-sib individuals, or the GRM relationship coefficients were near 0.25 while those of the NRM were near 0 as would be the case for half-sibs mistakenly recorded as unrelated. After elimination of these suspect individuals, the correlation coefficient between NRM and GRM increased from 0.856 to 0.866. Finally, 988 genotyped individuals remained for genomic prediction with a mean MAF of 0.243 and mean observed heterozygosity of 0.326.

Additional carcass records for 1438 non-genotyped progeny-tested steers were collected from the 39th to the 51st selection batches for the single-step and PBLUP analyses. Ancestors of the 2426 individuals with carcass records contributed to an 11-generation pedigree file that included 9637 animals.

Genotyped individuals were assigned to five mutually exclusive groups for cross-validation. K-means clustering based on pedigree relationship coefficients was used to minimize the relatedness between training and validation sets [13]. The five groups included 172, 280, 199, 139 and 198 individuals, respectively. Each group was used as the validation set while the remaining genotyped individuals were included in the training set. In SSGBLUP, SSBR and PBLUP with phenotypes on all animals, non-genotyped individuals were included in the training set. Phenotypes were pre-adjusted for contemporary group and age effects using multiple-trait PBLUP because animals from some progeny-test batches were assigned to different groups and because some analyses included additional non-genotyped animals from the same batches as genotyped animals.

### Single-trait statistical models

#### Pedigree-based BLUP

In these analyses, the adjusted phenotypes were modeled as:

$${\mathbf{y}} = {\mathbf{1}}\upmu + {\mathbf{Zu}} + {\mathbf{e}},$$

where **y** is a vector of adjusted phenotypic records from *n*_*y*_ animals, **1** is a vector of 1s, μ is the overall mean, **Z** is the design matrix allocating records to breeding values, **u** is the vector of breeding values, **e** is a random vector of residuals. It was assumed that ${\mathbf{u}} \sim N({\mathbf{0}},{\mathbf{A}}\sigma_{g}^{2} )$, where **A** is the numerator relationship matrix and *σ*_*g*_^2^ is the additive genetic variance. Residuals were assumed to be independently and identically distributed (iid) with null means and variance *σ*_*e*_^2^. Pedigree-based BLUP with phenotypes either on all animals or only on genotyped animals were referred to as PBLUP (*n*_*y*_ = 2426 minus validation animals*)* and PBLUP-G (*n*_*y*_ = 988 minus validation animals), respectively. Adjusted phenotypes were used to account for fixed effects in the validation set.

#### Bayesian methods using only genotyped animals

In these analyses, the adjusted phenotypes were modeled as:

$${\mathbf{y}} = {\mathbf{1}}\upmu + {\mathbf{M}}_{\text{g}} {\varvec{\upalpha}} + {\mathbf{e}}\text{,}$$

where **y**, **1** and **e** are *n*_*y*_ × 1 vectors for *n*_*y*_ = 988 minus genotyped validation animals, μ is as defined before, **M**_g_ is the *n*_*y*_ × *p* matrix of SNP covariates at *p* loci, and **α** is a *p* × 1 random vector of allele substitution effects. A flat prior was used for μ. The prior for **e** was ${\mathbf{e}}|\upsigma_{\text{e}}^{2} \sim{\text{N}}(0,{\mathbf{I}}\upsigma_{\text{e}}^{2} )$ with $(\upsigma_{\text{e}}^{2} |\upnu_{\text{e}} ,\text{S}_{\text{e}}^{2} ) \sim\upnu_{\text{e}} \text{S}_{\text{e}}^{2}\upchi_{{\upnu_{\text{e}} }}^{2}$. Priors for SNP effects were a mixture of a point mass at zero and a t-distribution in BayesB or a mixture of a point mass at zero and a normal distribution conditional on a common variance of SNP effects in BayesC and BayesC*π* methods [2]. These methods were referred to as BayesB, BayesC or BayesCπ, and ignored adjusted phenotypes on non-genotyped animals, as for PBLUP-G.

#### Single-step GBLUP

In the single-step GBLUP analyses, the adjusted phenotypes were modeled as:

$${\mathbf{y}} = {\mathbf{1}}\upmu + {\mathbf{Zu}} + {\mathbf{e}},$$

where **y** is the vector of adjusted phenotypes as before except that it includes both genotyped and non-genotyped individuals i.e. *n*_*y*_ = 2426 minus validation animals, μ and **e** are as defined before, with residuals that are iid with null means and variance *σ*_*e*_^2^, **Z** is the design matrix allocating records to breeding values, **u** is the vector of breeding values for both genotyped and non-genotyped individuals but now ${\mathbf{u}} \sim N({\mathbf{0}},{\mathbf{H}}\sigma_{g}^{2} )$, where:

$${\mathbf{H}} = \left[ {\begin{array}{*{20}c} {{\mathbf{A}}_{\text{ng}} {\mathbf{A}}_{\text{gg}}^{ - 1} {\mathbf{GA}}_{\text{gg}}^{ - 1} {\mathbf{A}}_{\text{gn}} + \left( {{\mathbf{A}}_{\text{nn}} - {\mathbf{A}}_{\text{ng}} {\mathbf{A}}_{\text{gg}}^{ - 1} {\mathbf{A}}_{\text{gn}} } \right) } & {{\mathbf{A}}_{\text{ng}} {\mathbf{A}}_{\text{gg}}^{ - 1} {\mathbf{G}}} \\ {{\mathbf{GA}}_{\text{gg}}^{ - 1} {\mathbf{A}}_{\text{gn}} } & {\mathbf{G}} \\ \end{array} } \right],$$

and **A**_gg_ is the 988 order partition of the numerator relationship matrix **A** that corresponds to genotyped animals, **A**_nn_ is the 11,075 order partition of **A** that corresponds to non-genotyped animals, **A**_ng_ or **A**_gn_ are partitions of **A** corresponding to relationships between non-genotyped and genotyped animals or vice versa, and **G** is a GRM of order 988. We applied three methods to construct the GRM. The standard **G** was constructed as ${\mathbf{G}} = \frac{{{\mathbf{TT}}^{\prime } }}{{\sum 2q_{i} (1 - q_{i} )}}$ (SSGBLUP-I) with **T** being the centered matrix of SNP covariates (${\mathbf{T}} = {\mathbf{M}}_{\text{g}} - \frac{1}{n}{\mathbf{11}}^{\prime } {\mathbf{M}}_{\text{g}}$), *q*_*i*_ representing the allele frequency of the *i*th SNP. This is the same **G** as previously used to compare relationship coefficients between NRM and GRM and eliminate the 19 individuals with genotype-pedigree conflicts, except that 19 rows and corresponding columns were deleted. In the standard **G**, the additive genetic variance attributed to each SNP genotype is equally important and GRM are identical for all traits. Recently, methodologies for constructing **G** with weighting factors to account for locus-specific variances were proposed [14–16]. The method reported by Wang et al. [14] calculates SNP effects from the solution of SSGBLUP-I and then reconstructs a new GRM using weights that are obtained from the previously calculated SNP effects. This can be repeated iteratively to obtain a sequence of GRM. In this approach, GRM will differ for each trait.

The prediction model based on the GRM constructed from one iteration was referred to as SSGBLUP-II and the GRM constructed from five iterations was referred to as SSGBLUP-III. To remove singularity, GRM can be blended with NRM [17] but this was not done in our study, nor were residual polygenic effects separately modeled in either SSGBLUP or SSBR. Instead, diagonal and off-diagonal elements of **G** were separately scaled so that their means equal the corresponding means of **A**_gg_, which is expected to remove the singularity of GRM in SSGBLUP that is introduced by centering the SNPs.

#### Single-step Bayesian regression methods

In the single-step Bayesian regression analyses, the adjusted phenotypes were modeled as:

$${\mathbf{y}} = {\mathbf{X\varvec\upbeta }} + {\mathbf{ZM\varvec\upalpha }} + {\mathbf{Z}}_{\text{n}} {\varvec{ \upepsilon }} + {\mathbf{e}},$$

where **y** is the adjusted phenotypic vector for both genotyped and non-genotyped individuals, $${\mathbf{X}} = \left[ {\begin{array}{*{20}c} {\mathbf{1}} & { - {\mathbf{Z}}_{\text{n}} {\mathbf{A}}_{\text{ng}} {\mathbf{A}}_{\text{gg}}^{ - 1} {\mathbf{1}}} \\ {\mathbf{1}} & { - {\mathbf{Z}}_{\text{g}} {\mathbf{1}}} \\ \end{array} } \right],\;{\varvec{\upbeta}} = \left[ {\begin{array}{*{20}c}\upmu \\ {\upmu_{\text{g}} } \\ \end{array} } \right],$$ μ is the overall mean, and μ_g_ represents the difference in breeding values between genotyped and non-genotyped animals, **Z** is the design matrix, $${\mathbf{M}} = \left[ {\begin{array}{*{20}c} {\widehat{{{\mathbf{M}}_{\text{n}} }}} \\ {{\mathbf{M}}_{\text{g}} } \\ \end{array} } \right],$$ where **M**_g_ is the matrix of SNP covariates for genotyped animals and $\widehat{{{\mathbf{M}}_{\text{n}} }} = {\mathbf{A}}_{\text{ng}} {\mathbf{A}}_{\text{gg}}^{ - 1} {\mathbf{M}}_{\text{g}} ,$ representing imputed SNP covariates for non-genotyped animals that are derived from genotyped relatives, ${\varvec{\upepsilon}}$ is the imputation residual, **Z**_n_ and **Z**_g_ are the design matrices allocating records to breeding values of non-genotyped animals and genotyped animals. Flat priors were used for μ and μ_g_. The prior for *e*_*i*_ is $e_{i} |\sigma_{e}^{2} \sim_{iid} N(0,\sigma_{e}^{2} )$ with $(\sigma_{e}^{2} |\nu_{e} ,S_{e}^{2} ) \sim \nu_{e} S_{e}^{2} \chi_{{\nu_{e} }}^{2}$. The prior for ${\varvec{\upepsilon}}$ is ${\varvec{\upepsilon}}|\sigma_{g}^{2} \sim N(0,({\mathbf{A}}_{\text{nn}} - {\mathbf{A}}_{\text{ng}} {\mathbf{A}}_{\text{gg}}^{ - 1} {\mathbf{A}}_{\text{gn}} )\sigma_{g}^{2} )$ with $(\sigma_{g}^{2} |\nu_{g} ,S_{g}^{2} ) \sim \nu_{g} S_{g}^{2} \chi_{{\nu_{g} }}^{2}$. The same priors for SNP effects as in BayesB, BayesC and BayesC*π* were used in single-step Bayesian regression methods and were referred to as SSBR-B, SSBR-C, or SSBR-C*π*.

The *π* values in the subsequent analyses for BayesB, BayesC, SSBR-B and SSBR-C were chosen such that they provided the highest accuracies from fivefold cross-validation. Accuracies in BayesB and BayesC were compared using various *π* values i.e. 0.9999, 0.999, 0.995, 0.99, 0.98 and, then, in steps from 0.95 to 0.6 decreasing by 0.05.

Analyses were performed with GenSel [5] for BayesB, BayesC and BayesC*π* methods using only genotyped animals. Estimated breeding values of PBLUP and SSGBLUP were obtained using the software BLUPF90 [18] modified for genomic analyses [17]. For SSBR methods, JWAS the Julia package for whole-genome analyses [19] was used.

### Validation

For each validation set, prediction accuracy was calculated as the correlation between the vector of adjusted phenotypes and the vector of estimated breeding values, divided by the square root of trait heritability. Prediction accuracies from these fivefold cross-validation sets were pooled to obtain a single prediction accuracy that was relevant to the method and trait by weighting each of the five validation correlations by the number of individuals in that set. Regressions of adjusted phenotype on estimated breeding value were calculated for all prediction methods.

### Genome-wide association studies

Genome-wide association studies (GWAS) were performed using the BayesB method with the *π* value that had given the highest prediction accuracy, in order to describe the genetic architecture for different traits in terms of window variance [20].

## Results

**Fig. 1:**
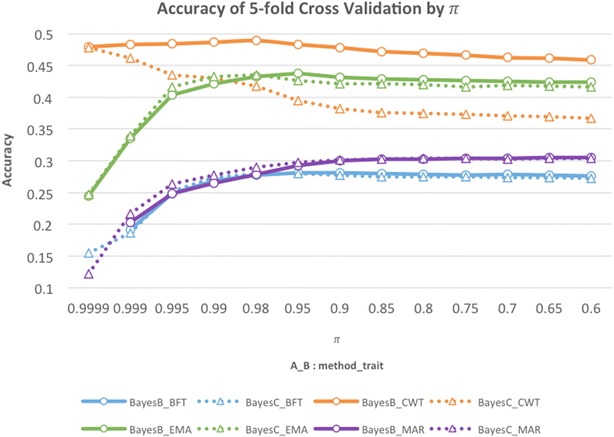
Fivefold cross-validation accuracies obtained with BayesB or BayesC using various assumed values for *π*

**Fig. 2:**
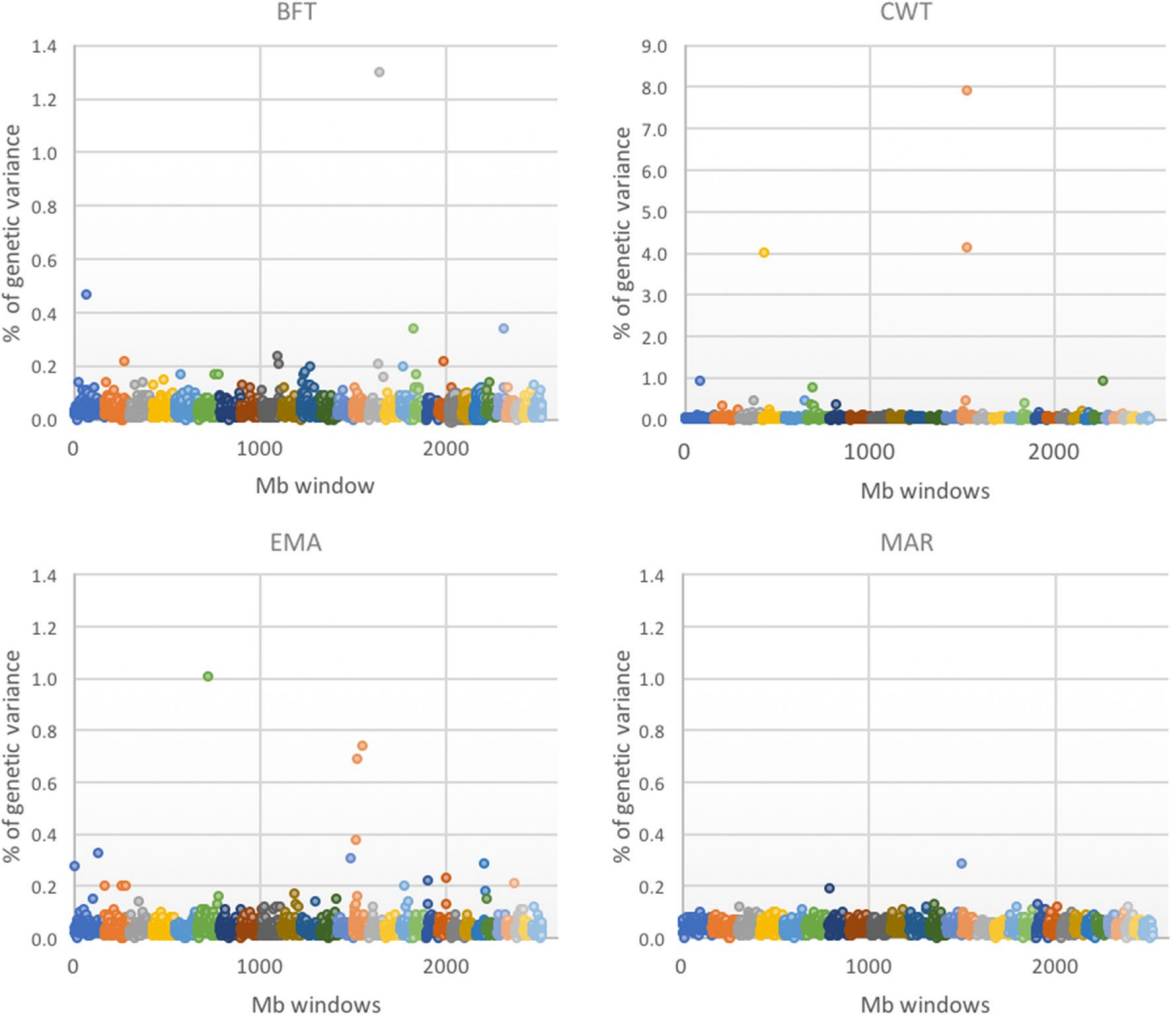
Results of the GWAS for each of the four traits. *Different colors* represent different autosomes (ordered from 1 to 29)

Predictive accuracies for the four traits obtained with BayesB and BayesC for different *π* values are in Fig. 1. For BFT, EMA and MAR, predictive accuracies of BayesB and BayesC were similar, but decreased as *π* increased, and fewer SNPs were assumed to have non-zero effects. For CWT, we observed a different pattern with accuracies increasing as *π* increased and accuracies of BayesB being always higher than those of BayesC. These two results suggest that CWT is influenced by a few quantitative trait loci (QTL) that explain a large proportion of the genetic variance. The proportions of genetic variance explained by 1-Mb non-overlapping genomic windows are in Fig. 2, and demonstrate that the QTL for CWT were larger than those for the other traits.

The *π* values that maximized the cross-validation accuracies in BayesB were 0.95, 0.98, 0.95, and 0.6 for BFT, CWT, EMA and MAR, respectively, and were used in SSBR-B. The corresponding *π* values in BayesC were 0.98, 0.9999, 0.98, and 0.6 for BFT, CWT, EMA and MAR, respectively, and were used in SSBR-C.

Several windows showed distinctly larger effects than the rest of the genome for BFT and EMA, but the window with the largest effect explained only about 1% of the genetic variance. For MAR, the windows showed smaller effects than those for BFT and EMA with the most significant window explaining less than 0.3% of the genetic variance. These results show that, for BFT, EMA and MAR, many QTL each with a small effect are widely distributed across the whole genome, which is consistent with the infinitesimal model. In contrast, for CWT, one window on chromosome 4 and two windows on chromosome 14 explained together more than 15% of the genetic variance while the other windows showed small effects. Using single-SNP association tests, Lee et al. [21] found similar results that indicated that SNPs on chromosome 14 were strongly associated with CWT in Hanwoo beef cattle. These differences in genomic architecture between the four traits probably explain the difference in the pattern of prediction accuracy between CWT and the three other traits as shown in Fig. 1. BayesB, which shrinks QTL with small effects to a greater extent than BayesC, may capture QTL with large effects better and therefore yield higher prediction accuracies [22]. BayesB and BayesC methods with a high *π* value tend to capture the same few QTL with large effects, thus their similar prediction accuracies.

Prediction accuracies of models SSGBLUP-I and SSBR-C (*π* = 0) without estimated variances were identical and equal to 0.351 for BFT, 0.415 for CWT, 0.413 for EMA and 0.377 for MAR as expected since these models with assumed variance parameters are equivalent in terms of prediction of breeding values [7]. In practice, variance components are often treated as unknown and are estimated in a separate analysis, e.g. restricted maximum likelihood (REML) followed by GBLUP, or jointly with an informative prior, e.g. BayesB, SSBR-B, etc. The variances of additive genetic effects, SNP effects and residual effects were estimated in the subsequent analyses described below.

**Fig. 3:**
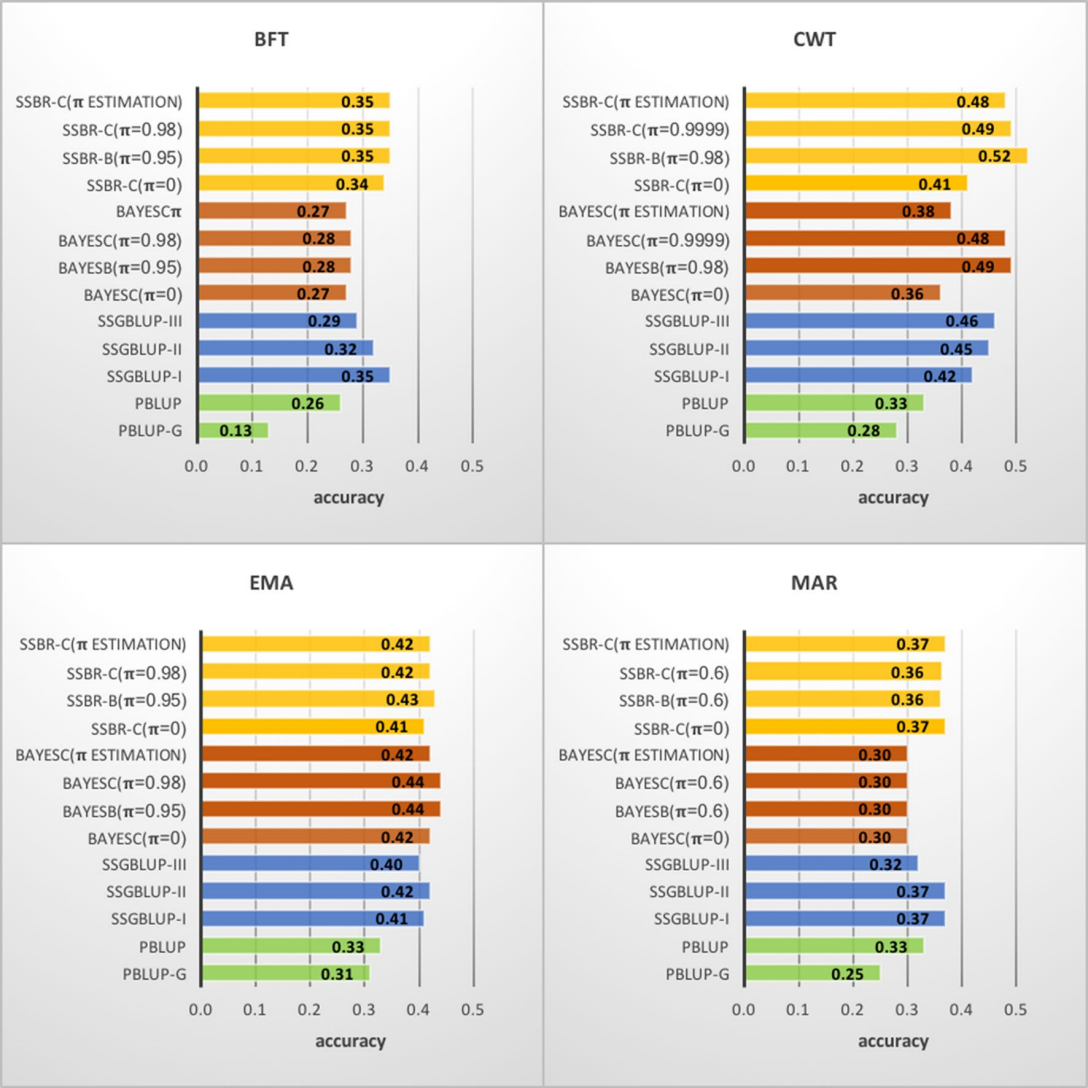
Prediction accuracies by cross-validation for a variety of methods applied to backfat (BFT), carcass weight (CWT), eye-muscle area (EMA) and marbling (MAR). Conventional PBLUP based on only genotyped individuals (PBLUP-G) or using all animals (PBLUP), BayesB with chosen *π* (BAYESC(*π* = chosen value)), BayesC with chosen *π* (BAYESC (*π* = chosen value)) BayesC with *π* = 0 (BAYESC (*π* = 0)) or BayesC estimating *π* (BAYESC (*π* ESTIMATION)), single-step genomic BLUP constructing two different genomic relationship matrix (SSGBLUP-I and SSGBLUP-II) and single-step Bayesian regression corresponding to Bayesian methods (SSBR-B (*π* = chosen value), SSBR-C (*π* = chosen value), SSBR-C (*π* = 0), and SSBR-C (*π* ESTIMATION))

To compare methods that use all individuals with those that use only genotyped individuals, prediction accuracies (Fig. 3) were calculated using PBLUP (all animals) and PBLUP-G (PBLUP using only phenotypes on genotyped animals), BayesB, BayesC, BayesC (*π* = 0), and BayesC*π*, SSGBLUP-I and SSGBLUP-II and SSBR-B, SSBR-C, SSBR-C (*π* = 0), and SSBR-C*π*.

### Genomic methods versus pedigree-based BLUP

Methods using genomic information always outperformed PBLUP with the same phenotypic data. Using data from only genotyped animals, accuracies were higher with BayesB, BayesC and BayesC*π* than with PBLUP-G for all traits. When data from both genotyped and non-genotyped individuals were used, prediction accuracies of the single-step methods were higher than those of PBLUP for all traits.

### Single-step methods versus BayesB, BayesC and BayesC*π*

For BFT and MAR, prediction accuracies of the single-step methods were higher than those of BayesB, BayesC and BayesC*π*. Gains in accuracy with the single-step methods ranged from +0.06 to +0.09 for BFT and from +0.05 to +0.07 for MAR, whereas for EMA, there was no advantage and only a slight gain in accuracy was observed in PBLUP versus PBLUP-G. For CWT, SSBR always outperformed the corresponding Bayesian methods using only genotyped individuals and the gains in accuracy were +0.05 (SSBR-C (*π* = 0) vs. BayesC (*π* = 0)), +0.01 (SSBR-C (*π* = 0.9999) vs. BayesC (*π* = 0.9999)), +0.10 (SSBR-C*π* vs. BayesC*π*) and +0.04 (SSBR-B (*π* = 0.98) vs. BayesB (*π* = 0.98)). However, although information from non-genotyped individuals was incorporated, for CWT prediction accuracy of SSGBLUP was lower than that of BayesC (*π* = 0.9999) and BayesB (*π* = 0.98) due to the benefits of mixture priors of the SNP effects for this particular trait.

### Comparisons between single-step methods

The differences in accuracies between single-step methods (yellow and blue bars in Fig. 3) were small for BFT, EMA and MAR, and a similar pattern was found between Bayesian methods (red bars in Fig. 3) using only genotyped individuals. For the CWT trait for which the GWAS detected a small number of regions with large effects, prediction accuracies differed with the method used. With the benefits of mixture priors and information from non-genotyped individuals, prediction accuracies of the SSBR methods, especially SSBR-B, were higher (+0.09) than those of the SSGBLUP methods. As for the SSBR methods with mixture priors, the SSGBLUP methods, which use weighted GRM (SSGBLUP-II and SSGBLUP-III), showed higher accuracies than SSGBLUP-I for CWT. Prediction accuracy of SSGBLUP-II was similar to that of SSGBLUP-I for EMA and MAR but lower for BFT. Prediction accuracy of SSGBLUP-III was lower than that of SSGBLUP-I for EMA, MAR and BFT. Regressions of adjusted phenotype on estimated breeding value did not show large differences among methods, but SSGBLUP-II and SSGBLUP-III had the lowest coefficients for all traits, much lower than 1, which indicates that their genomic predictions are biased upwards (Table 1).

**Table 1:** Regression coefficient of adjusted phenotype on estimated breeding values for backfat (BFT), carcass weight (CWT), eye-muscle area (EMA) and marbling (MAR) traits

Prediction methods	Trait			
	BFT	CWT	EMA	MAR
SSBR-C (*π* estimation)	0.85	0.97	0.99	0.88
SSBR-B (*π* = chosen)^a^	0.88	1.08	1.07	0.74
SSBR-C (*π* = chosen)^b^	0.88	1.02	1.04	0.89
SSBR-C (*π* = 0)	0.86	1.21	1.00	0.87
BayesC (*π* estimation)	0.82	1.05	1.05	0.86
BayesB (*π* = chosen)^a^	0.82	1.03	1.26	0.70
BayesC (*π* = chosen)^b^	0.88	1.06	1.12	0.87
BayesC (*π* = 0)	0.86	1.20	1.09	0.88
SSGBLUP-I	0.73	1.15	0.97	0.79
SSGBLUP-II	0.54	0.84	0.75	0.64
SSGBLUP-III	0.52	0.90	0.79	0.61
PBLUP	0.76	1.12	1.02	0.93
PBLUP-G	0.61	1.33	1.30	0.92

^a^Chosen *π* of BayesB and SSBR-B for BFT, CWT, EMA and MAR were 0.95, 0.98, 0.95 and 0.6, respectively

^b^Chosen *π* of BayesC and SSBR-C for BFT, CWT, EMA and MAR were 0.98, 0.9999, 0.98 and 0.6, respectively

## Discussion

Prediction accuracies of all methods using genomic information were higher than those of pedigree-based BLUP. However, the degree of superiority of genomic selection differed between methods and traits.

We hypothesize that the advantage of including phenotypic observations from non-genotyped animals into an analysis using phenotypic observations from genotyped animals would be similar for pedigree methods (PBLUP compared to PBLUP-G) and for genomic methods (SSBR-C compared to BayesC). Simultaneous use of all pedigree, phenotypic and genomic information in single-step methods improved prediction accuracy relative to methods that only use data from genotyped animals for all traits, except EMA. For EMA, there was similarly little benefit from including the extra data in the PBLUP analyses (compared to PBLUP-G).

Both SSBR and SSGBLUP methods showed similar prediction accuracies when the genetic architecture appeared to approach the infinitesimal model as was the case for BFT, EMA, and MAR. However, for CWT, prediction accuracies of the SSBR methods were higher than those of SSGBLUP when there were only a few QTL with large effects. For that trait, the SSBR methods benefited from the use of the mixture priors.

The largest benefit of the SSBR methods was reached when an appropriate *π* was applied. However, it is computationally intensive to find this value of *π* through cross-validation. Methods for estimating *π* are beneficial, but they require large datasets. An appropriate *π* was more critical for the Bayesian methods that only used genotyped individuals than for the SSBR methods. For example, differences in prediction accuracies between BayesC (*π* = 0.9999) and BayesC*π* reached values of 0.10 but only of 0.01 between SSBR-C (*π* = 0.9999) and SSBR-C*π*. Presumably, priors become less important in the single-step analyses where more data are used.

Three factors can result in increased accuracy. First, the inclusion of genomic information, which was revealed when genomic methods were compared to pedigree-based BLUP. Second, the use of additional phenotypic information from including non-genotyped individuals, which was shown by comparing Bayesian methods using only genotyped animals with their corresponding single-step methods. Third, the use of methods that exploit genomic regions with large effects, as was found for one of the four traits using either mixture priors or iterative weighted methods for computing GRM.

SSGBLUP with iterative calculation of weighted genomic matrices had the disadvantage that it reduced prediction accuracy and increased bias for traits that were not associated with genomic regions with large effects, whereas the Bayesian models with mixture priors performed comparably regardless of the genomic architecture. SSGBLUP with iterative calculation of weighted genomic matrices shrinks small effects to zero, and more so with each additional iteration. There is no statistical basis to determine the optimal number of iterations except by trial and error, and neither one nor five iterations resulted in improvements in this dataset.

In this study, which is based on a small population of Hanwoo cattle, prediction accuracy was higher for all genomic evaluations compared to pedigree-based BLUP. In such a situation, where the genomic reference population is relatively small, single-step methods, which can routinely account for genomic regions with large effects when they are present, are recommended for additional gains in accuracy.

## Conclusions

The “single-step” methodologies, which take advantage of all pedigree, phenotypic and genomic information simultaneously, give similar or higher prediction accuracies compared to methods using only genotyped individuals. Compared to SSGBLUP, the SSBR methods showed additional benefit for the CWT trait, which is associated with QTL with large effects. There is no disadvantage in using SSBR methods for all traits.
